# Targeted integration of EpCAM-specific CAR in human induced pluripotent stem cells and their differentiation into NK cells

**DOI:** 10.1186/s13287-021-02648-4

**Published:** 2021-11-21

**Authors:** Shin Yi Tang, Shijun Zha, Zhicheng Du, Jieming Zeng, Detu Zhu, Yumei Luo, Shu Wang

**Affiliations:** 1 Department of Biological Sciences, National University of Singapore, 14 Science Drive 4, Singapore, 117543 Singapore; 2 Institute of Bioengineering and Nanotechnology, Singapore, 138669 Singapore; 3 Key Laboratory for Major Obstetric Diseases of Guangdong Province, The Third Affiliated Hospital of Guangzhou Medical University, Guangzhou, 510150 Guangdong China

**Keywords:** Induced pluripotent stem cells (iPSC), Natural killer cells (NK), Chimeric antigen receptors (CAR), Adeno-associated virus integration site 1 (AAVS1), Zinc finger nuclease (ZFN), Genetic engineering, Targeted integration, NK differentiation, Epithelial cell adhesion molecule (EpCAM), Immunotherapy

## Abstract

**Background:**

Redirection of natural killer (NK) cells with chimeric antigen receptors (CAR) is attractive in developing off-the-shelf CAR therapeutics for cancer treatment. However, the site-specific integration of a CAR gene into NK cells remains challenging.

**Methods:**

In the present study, we genetically modified human induced pluripotent stem cells (iPSCs) with a zinc finger nuclease (ZFN) technology to introduce a cDNA encoding an anti-EpCAM CAR into the adeno-associated virus integration site 1, a “safe harbour” for transgene insertion into human genome, and next differentiated the modified iPSCs into CAR-expressing iNK cells.

**Results:**

We detected the targeted integration in 4 out of 5 selected iPSC clones, 3 of which were biallelically modified. Southern blotting analysis revealed no random integration events. iNK cells were successfully derived from the modified iPSCs with a 47-day protocol, which were morphologically similar to peripheral blood NK cells, displayed NK phenotype (CD56+CD3-), and expressed NK receptors. The CAR expression of the iPSC-derived NK cells was confirmed with RT-PCR and flow cytometry analysis. In vitro cytotoxicity assay further confirmed their lytic activity against NK cell-resistant, EpCAM-positive cancer cells, but not to EpCAM-positive normal cells, demonstrating the retained tolerability of the CAR-iNK cells towards normal cells.

**Conclusion:**

Looking ahead, the modified iPSCs generated in the current study hold a great potential as a practically unlimited source to generate anti-EpCAM CAR iNK cells.

**Supplementary Information:**

The online version contains supplementary material available at 10.1186/s13287-021-02648-4.

## Background

Natural killer (NK) cells have been attractive in adoptive cell-based cancer immunotherapy owing to their advantages including innate surveillance of tissue abnormality, low risk of cytokine release syndrome and readiness in allogenic usage [1, 2]. NK cells, as innate lymphoid cells, mount immune response against cancer cells through an array of germline-encoded activating and inhibitory receptors [2]. While the intrinsic anti-tumour capacity of NK cells is intriguing in treating haematological malignancies, their efficacy in treating solid tumours has yet to be established [3]. To enhance the anti-tumour activity and specificity of NK cells in solid tumour treatment, chimeric antigen receptors (CAR) are introduced to generate CAR-expressing NK cells for advanced cancer therapy [4–6].

However, unlike the success and feasibility in CAR-T cell generation, CAR-NK cell generation from primary NK cells is faced with key obstacles such as the cost- and time-consuming expansion of primary NK cells and the relatively low efficiency of genetic modification [5–9]. To address these challenges, induced pluripotent stem cells (iPSCs) have been tested as an unlimited cell source for NK cell generation [10–13]. It has also been reported that a CAR can be introduced into iPSCs by lentiviral vector transduction [14] and electroporation-based transposition [15] to facilitate CAR-NK generation. Although efficient, viral transduction and transposon-based CAR introduction randomly integrate transgene into the genome, posing the potential risk of functional gene disruption and genome instability in engineered CAR-expressing NK cells.

Genetic modification of iPSCs with site-specific gene editing tools has been intensively studied from ZFNs and TALENs to CRISPR/Cas system over the last decade [16, 17] and it was also well-established in our laboratory [18–20]. With the advantage of site-specific gene editing tools like ZFNs, CAR transgene should be able to be specifically integrated into a designated safe gene locus in iPSCs with controllable copy number and potent expression. In this study, we tested whether a validated ZFN gene editing tool could be used to introduce a CAR gene into the AAVS1 locus in iPSCs in a site-specific integration manner. More importantly, we examined whether the CAR gene-modified iPSCs were capable of differentiating into CAR-expressing NK cells.

We chose a CAR specific to EpCAM for this study. EpCAM, a cell surface protein, is over-expressed on a variety of epithelial-derived carcinomas, including adenocarcinomas of colon, stomach, pancreas, lung, ovarian, and breast and plays important roles in modulating cell adhesion and signalling pathways in cancers [21]. As such, EpCAM is attractive for targeted cancer therapy.

## Methods

### Cell culture

Human peripheral blood mononuclear cell (PBMC)-derived iPSC lines were generated as reported previously [11, 22]. iPSCs were cultured with mTeSR1 (StemCell Technologies, Vancouver, BC, Canada) on six-well plates coated with Matrigel (BD Biosciences, Franklin Lakes, NJ) and mechanically passaged every 7 days by treating with 1 mg/ml Dispase (StemCell Technologies) at 37 °C for 5 min. Murine bone marrow-derived stromal cell line OP9-DLL1 (Riken BRC Cell Bank, Ibaraki, Japan) was cultured in Minimum Essential Medium α (MEM α) (Gibco, Waltham, MA) supplemented with 20% foetal bovine serum (FBS, Gibco). Tumour cell lines including breast ductal carcinoma cell line BT474 (ATCC HTB-20), breast metastatic carcinoma cell line MDA-MB-453 (ATCC HTB-131), breast adenocarcinoma cell line MCF7 (ATCC HTB-22) and breast ductal metastatic carcinoma cell line MDA-MB-435S (ATCC HTB-129) were cultured as recommended by ATCC. Primary NK cells were expanded from fresh PBMCs by co-culturing with inactivated modified K562 feeder cells as described previously [23]. NK cells were harvested as population of over 90% CD3-CD56+ cells after the co-culture.

### Plasmid construction

**Fig. 1:**
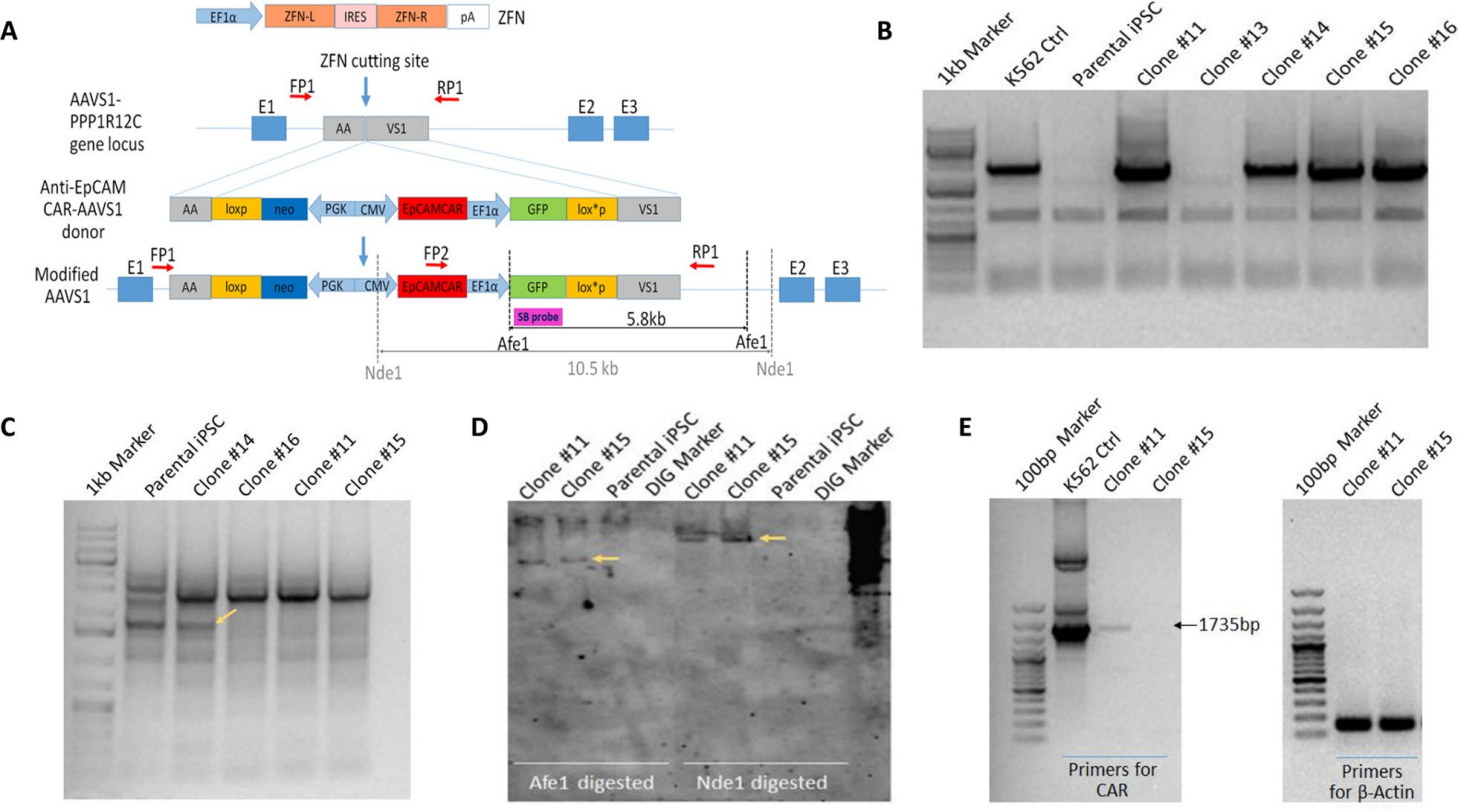
Genetic modification to introduce anti-EpCAM CAR into iPSCs. **A** Schematic diagram showing ZFN-mediated AAVS1 site integration of anti-EpCAM CAR. The anti-EpCAM CAR-AAVS1 donor, the cutting site of ZFN, the modified AAVS1 following homologous recombination, the site of primers (FP1, FP2, RP1) binding and Southern Blot probe (SB probe) are shown. Green fluorescence protein (GFP) and neomycin resistance (neo) genes are included in the plasmid to facilitate the clone selection. **B** PCR genotyping demonstrating AAVS1 integration. After the nucleofection of iPSCs and geneticin (G418) selection for 2 weeks, 4 out of 5 clones were found to contain the insertion in AAVS1. A K562 EGFP-AAVS1 stable clone was used as the control to show AAVS1 site modification. **C** PCR genotyping demonstrating 3 out of 4 clones being biallelically modified. Arrow: A 1.7 kb fragment is amplified from the unmodified allele. **D** Southern Blot analysis demonstrating specific AAVS1 site integration in 2 clones without other non-specific bands. Arrows: A single 10.5 kb band with Nde1 digestion and a single 5.8 kb band with Afe1 digestion. **E** RT-PCR demonstrating the weak, but detachable expression of anti-EpCAM CAR in one of the two modified iPSCs clones

The third-generation anti-EpCAM CAR was designed previously in laboratory [24] consisting of an scFv of 4D5MOC-B humanized mAb, CD8 alpha hinge and transmembrane domain (uniprot P01732, residues 128–210), CD28 co-stimulatory domain (uniprot P10747, residues 180–220), 4-1BB co-stimulatory domain (uniprot Q07011, residues 214–255) and the CD3zeta intracellular ITAM domain (uniprot P20963, residues 52–164). To construct the donor vector for AAVS1 site homologous recombination, the complete sequence of a cytomegalovirus (CMV) promoter driving the expression of anti-EpCAM CAR from the mRNA CAR vector [24] was subcloned into pFB-EF1α-EGFP-AAVS1 donor vector developed previously [18] containing a eukaryotic translation elongation factor 1 alpha (EF1α) promoter driving GFP expression cassette and a mouse phosphoglycerate kinase 1 (PGK) promoter driving the neomycin resistant gene expression cassette, which was flanked by homologous sequence of AAVS1 locus (Fig. 1A).

### Generation of CAR-modified iPSCs

Previously, our group has successfully and efficiently engineered fibroblast-derived iPSCs with ZFNs-mediated AAVS1 site targeting [18]. Using the same method, we generated genetically modified iPSCs with AAVS1 site-specific integration of anti-EpCAM CAR. Five μg of anti-EpCAM CAR-AAVS1 donor and 5 μg of zinc-finger nuclease (ZFN) vector [18] were electroporated into iPSCs through nucleofection using Human Stem Cell Nucleofector Kit 1 (Lonza, Basel, Switzerland, VPH-5012) and program B-016 of the Lonza NucleofectorTM 2b device. After nucleofection, the cells were seeded directly onto Matrigel-coated six-well plates and cultured with mTeSR1. Each GFP-positive clone was manually picked and transferred to separate wells after one week. Subsequently, the cells were maintained in 25 μg/ml geneticin (G418) drug selection medium to further enrich the GFP-positive population.

### Genotyping and reverse transcription polymerase chain reaction (RT-PCR)

Cells were harvested and their genomic DNA were isolated using DNeasy® Blood & Tissue Kit (Qiagen, Hilden, Germany) following manufacturer’s instruction. PCR genotyping was performed with KAPA HiFi Hotstart Readymix (KAPA Biosystem, Woburn, MA), 100 ng of genomic DNA, PCR primers AAVS1-HR-L2-F (5′-TCTAACGCTGCCGTGCCGTCTCTCTCCTGA-3′) and AAVS1-Neo-R (5′-ATATTGCTGAAGAGCTTGGCGGCGAA-3′) in thermocycler with the reaction of 95 °C for 3 min; 35 cycles of 98 °C for 20 s, 63.5 °C for 15 s, 72 °C for 90 s; and final extension of 72 °C for 5 min before holding at 4 °C. For monoallelic and biallelic detection, the three primers used as illustrated in Fig. 1A were FP1 (5′-CGGGGATGCAGGGGAACGGGGCTCAGTCTG-3′), FP2 (5′-GGTGACAAGCCTTCTGCTCTGTGAGTTACC-3′) and RP1 (5′-CTCTGCCCTCTAACGCTGCCGTCTCTCTCC-3′). The parameters of the thermocycler were set as 95 °C for 3 min; 35 cycles of 98 °C for 20 s, 62 °C for 15 s 72 °C for 2.5 min; and final extension of 72 °C for 5 min before holding at 4 °C. The amplicons were analysed on 1% agarose gel.

For RT-PCR of CAR mRNA expression, the total RNA from iPSCs was isolated with TRIZOL reagent (Invitrogen Life Technologies, Carlsbad, CA). cDNA was synthesized using the SuperScript III First-Strand Synthesis System (Invitrogen Life Technologies) and treated with DNase. Five ng of cDNA template was amplified with Platinum PCR SuperMix High Fidelity (Thermo Fisher Scientific, Waltham, MA) and primers CAR-F (5′-ATGCTTCTCCTGGTGACAAGC-3′) and CAR-R (5′-TCCTCTAGTACTTCTCGACAAGC-3′). The parameters of the thermocycler were set as 95 °C for 5 min; 35 cycles of 95 °C for 30 s, 50 °C for 15 s 72 °C for 90 s; and final extension of 72 °C for 5 min before holding at 4 °C. PCR-amplified products were visualized by gel electrophoresis with 2% agarose gel.

### Southern blotting

For Southern blot analysis, 15 μg of genomic DNA was digested with 15 μl of AfeI or NdeI restriction enzymes (New England BioLabs, Ipswich, MA) at 37 °C overnight. As a positive control, 0.5 ng of plasmid DNA was digested with 0.25 μl of AfeI or NdeI restriction enzymes at 37 °C for 2 h. Subsequently, the digested DNA was separated on 1% agarose gel at 20 Volt in cold room overnight. The DNA was then transferred to a nylon membrane provided in the iBlot ® DNA Transfer Stack (Invitrogen) with the pre-set program 8 of the iBlot Gel Transfer Device (Invitrogen) for 7 min. The membrane was denatured with 1.5 M NaCl/0.5 M NaOH solution for 10 min and air-dried for 10 min before cross-linking by ultraviolet light at 120 mJ/cm^2^ twice. The membrane was then pre-hybridized with DIG Easy Hyb (Roche Diagnostics, Indianapolis, IN) for 1 h and then hybridized overnight at the hybridization temperature of 48 °C with DIG-labelled probe. The probe was synthesized using the PCR DIG Probe Synthesis Kit (Roche Diagnostics). After hybridization, the membrane was washed and blocked using DIG Wash and Block Buffer Set (Roche Diagnostics) according to the manufacturer’s instructions. Lastly, the membrane was incubated with anti-digoxigenin-AP antibody for 1.5 h and developed with CDP-Star provided in the DIG DNA Labelling and Detection Kit (Roche Diagnostics) followed by visualization.

### Generation of iPSC-derived CAR-expressing NK (CAR-iNK) cells

To generate CAR-iNK cells from genetically modified iPSCs, a 47-day in vitro differentiation protocol was adopted as published previously [11]. For hematopoietic differentiation, iPSCs were co-cultured on the overgrown OP9 expressing Notch ligand delta-like 1 (OP9-DLL1) cells in OP9 medium (MEM α with 20% FBS). For lymphoid commitment, the iPSC/OP9 co-culture cells were harvested and further co-cultured with OP9-DLL1 cells in OP9 medium supplemented with 10 ng/ml stem cell factor (SCF) (Peprotech, Rocky Hill, NJ), 5 ng/ml Fms-related tyrosine kinase 3 ligand (FLT3L) (Peprotech), 5 ng/ml IL-7 (Peprotech) and 10 ng/ml IL-15 (Peprotech). Half medium was changed every 3 days. The differentiated cells were harvested with Versene (Gibco) and passaged to new feeder layer every week. CAR-iNK cells were harvested at day 47 of the differentiation.

### Flow cytometry analysis

For flow cytometry analysis, cells were harvested, washed and re-suspended in 100 μl of PBS (Gibco) supplemented with 1% FBS (Gibco). Antibodies were added and incubated at 4 °C for 20 min in the dark. Samples were then washed and analysed by a FACSCalibur flow cytometer (BD Biosciences). To examine CAR expression, cells were first stained with biotin-SP (long spacer) AffiniPure F(ab’)2 fragment goat anti-mouse IgG (115-066-072; Jackson Immunoresearch Laboratories, Bar Harbor, Maine) followed by allophycocyanin (APC)-conjugated streptavidin (016-130-084; Jackson). For phenotyping of NK cells, the following anti-human antigen fluorescent conjugated antibodies were used: anti-CD56-APC (BD 555518, BD Biosciences), anti-CD45-PC7 (BD 557748, BD Biosciences), anti-CD3-PE (130-091-374, Miltenyi Biotec, Bergisch Gladbach, Germany), anti-NKp46 (CD335)-PE (BD 557991, BD Biosciences), anti-NKp30 (CD337)-PE (BD 558407, BD Biosciences), anti-NKp44 (CD336)-PE (BD 558563, BD Biosciences), anti-NKG2D (CD314)-PE (BD 557940, BD Biosciences), anti-NKG2A(CD159a)-PE (IM3291U, Beckman Coulter, Brea, CA) and anti-CD94 (Kp43)-PE (IM2276, Beckman Coulter). For antigen detection, anti-CD326 (EpCAM)-APC (130-091-254, Miltenyi Biotec) and anti-HLA-ABC-APC (130-101-467, Miltenyi Biotec) were used.

### Cell cytotoxicity assay

The cytotoxicity of effector cells against target cells was examined by flow cytometry. Effector cells were co-cultured with 2 × 10^4^ target cells, which were prelabelled with 0.5 μM carboxyfluorescein diacetate succinimidyl ester (CFSE) (Thermo Fisher Scientific), at varying effector to target (E:T) ratios at 37 °C for 4 h. After incubation, co-culture samples were stained by 7-Amino-Actinomycin D (7-AAD) (BD Biosciences) on ice for 10 min in the dark. Samples were then washed and analysed by flow cytometry. Cytolytic effect on target cells was evaluated based on the percentage of 7-AAD stained population in CSFE-positive population.

## Results

### Generation of CAR-expressing iPSCs by ZFNs-mediated AAVS1 site-specific modification

To introduce the CAR expression cassette into iPSCs, we chose the reported “safe harbour”, AAVS1 site [25, 26], as the target locus for specific integration of a CAR transgene. First, we designed a donor sequence encoding a cytomegalovirus (CMV) promoter driven third-generation anti-EpCAM CAR, which was comprised of a humanized single-chain variable fragment (scFv) 4D5MOC-B [27], a CD8 alpha hinge transmembrane region, two co-stimulatory domains (CD28 and 4-1BB) and a CD3 zeta T cell activation domain, as shown in Additional file 1: Fig. S1. Green fluorescence protein (GFP) and neomycin-resistance (neo) genes were also included in the donor sequence to facilitate the clone selection. This donor sequence was flanked by AAVS1 site homologous sequences for site-specific integration (Fig. 1A).

Using a previously developed method [18], we electroporated ZFNs plasmid and the above donor plasmid together into the human PBMC-derived iPSCs. Electroporated iPSCs were subject to a two-stage selection procedure: (1) manual selection of GFP-positive regions one week after nucleofection, and followed by (2) geneticin (G418) drug treatment to enrich GFP-positive cell populations (Additional file 1: Fig. S2). Based on above selections, GFP positive iPSC clones were collected for subsequent analysis.

PCR genotyping was performed using genomic DNA extracted from the collected clones, showing four out of five selected clones bearing the alleles of AAVS1 site integration with the presence of a 2.4 kb amplicon (Fig. 1B). We next examined the allele of wild type AAVS1 by PCR in the four clones with integrations and detected that only one of the four clones showed the 1.7 kb wild type AAVS1 amplicon (Fig. 1C), indicating three iPSC clones with biallelic AAVS1 modification and one iPSC clone with monoallelic AAVS1 modification out of the five selected clones. The wild-type AAVS1 and EpCAM CAR amplicons were sequenced full length to confirm identity (Additional file 1: Fig. S3).

To verify the specificity of this ZFNs-mediated AAVS1 site integration, we examined two biallelic AAVS1-integrated iPSC clones #11 and #15 by Southern blot analysis using a probe specific for GFP gene. Only a single band was observed with either NdeI or AfeI digestion in both clones, whereas no fragment was observed in the unmodified parental iPSCs (Fig. 1D). These results indicated that the donor cassette was specifically integrated into the AAVS1 site without random integration in these two iPSC clones, which was consistent with the high specific integration rate of ZFN/AAVS1 system in iPSC modification [18].

With the AAVS1 site-specific integration of the CAR expression cassette, we next investigated the CAR expression in modified iPSC clones. We only detected weak expression of anti-EpCAM CAR gene in iPSC clone #11, while no expression was observed in clone #15 by RT-PCR (Fig. 1E). Thus, iPSC clone #11 was chosen for subsequent differentiation. Overall, we successfully achieved CAR-expressing human iPSC clone with ZFNs-mediated site-specific integration to introduce an anti-EpCAM CAR transgene into the AAVS1 locus.

### Generation of CAR-expressing iNK cells from genetically modified iPSCs

**Fig. 2:**
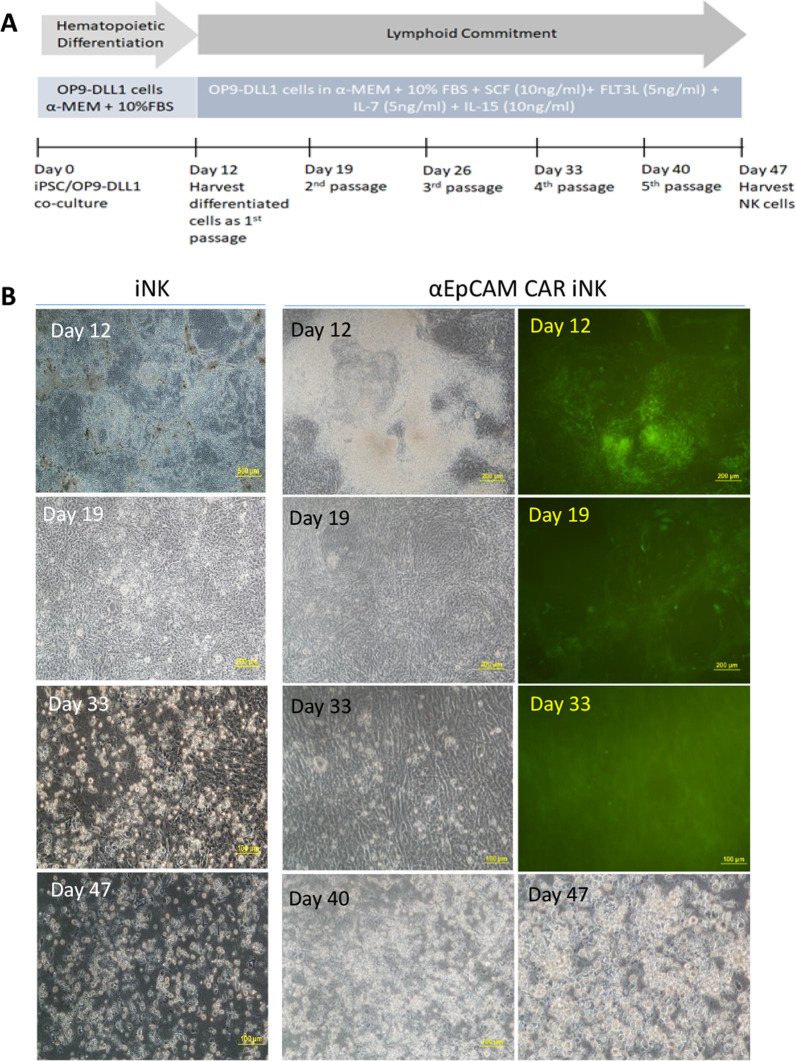
Differentiation of CAR-expressing iPSCs into iNK cells. **A** Schematic drawing of the two-step in vitro iPSC-NK differentiation scheme. **B** Cells morphology changes during the differentiation

Our previous study has revealed a two-stage protocol to generate NK cells from iPSCs [11]. Here we adopted this 47-day differentiation protocol to generate iPSC-derived NK (iNK) cells from CAR-expressing iPSCs. Both unmodified iPSCs and CAR-expressing iPSCs were subjected to this differentiation. In details, iPSCs were firstly co-cultured with OP9-DLL1, a modified OP9 cell line expressing Notch ligand Delta-like-1 (DLL1, Additional file 1: Fig. S4), for hematopoietic differentiation. On day 12, the differentiated cells were collected and co-cultured with OP9-DLL1 in the presence of interleukin 7 (IL-7), IL-15, stem cell factor (SCF), Fms-related tyrosine kinase 3 ligand (FLT3L) for lymphoid commitment. The cells were then passaged and seeded on fresh OP9-DLL1 every week until day 47 (Fig. 2A). Unmodified iPSCs and CAR-expressing iPSCs showed similar morphology during the 47 days of differentiation (Fig. 2B). After the 12 days of hematopoietic differentiation, the differentiated colonies of CAR-expressing iPSCs showed reduced GFP expression on the feeder layer, which could be the result of decreased expression driven by eukaryotic translation elongation factor 1 alpha (EF1α) promoter in differentiated cells comparing to its expression in pluripotent stem cells [28]. Morphologically, the differentiated cells gradually became small, bright, round, suspension cells from day 19 to day 40 indicating lymphoid commitment. Functionally, the differentiated cells started to kill feeder cells at the later stage of the differentiation. The number of suspension cells in the cell culture medium was obviously expanded from day 40 to day 47 (Fig. 2B). Both unmodified iPSCs and CAR-expressing iPSCs could yield similar numbers of suspension cells, assumed to be iNK cells, by harvest on day 47. We could usually obtain 15 × 10^6^ iNK cells from 3 × 10^6^ iPSCs seeded initially, with the highest yield of 100 × 10^6^ iNK cells from 4.5 × 10^6^ iPSCs in one experiment. The differentiated suspension cells were harvested for further analysis.

### Characterization of CAR-expressing iNK cells

**Fig. 3:**
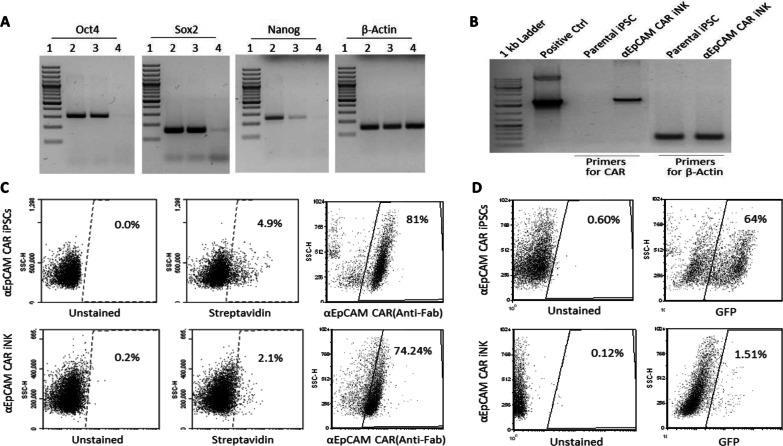
CAR expression after the differentiation of iPSCs into iNK cells. **A** RT-PCR analysis on the expression of pluripotent markers Oct4, Sox2 and Nanog in parental iPSCs (Lane 2), genetically modified iPSCs (Lane 3), and differentiated iNK cells (Lane 4). A 100-bp DNA ladder is included as a reference marker in Lane 1. **B** RT-PCR analysis showed that the expression of anti-EpCAM CAR was easily detectable after differentiation into iNK cells. **C** Flow cytometry analysis demonstrating anti-EpCAM CAR expression upon NK cell differentiation. The CAR expression was examined by staining with a biotinylated primary antibody against mouse IgG Fab fragment, followed by an APC-streptavidin conjugate. Unstained and APC-streptavidin conjugate-stained iPSCs and iNK cells are included as negative controls. **D** Flow cytometry analysis of GFP expression to demonstrate a diminished GFP expression in differentiated iNK cells

To evaluate the differentiation of iPSCs to iNK cells, pluripotent markers were examined by RT-PCR in iPSCs and differentiated cells. As showed in Fig. 3A, all three pluripotent markers, Oct4, Sox2 and Nanog, were detectable in unmodified iPSCs and CAR-expressing iPSCs but not observed in differentiated iNK cells. This indicated the fully differentiation of iPSCs after the 47-day protocol, while the CAR-expressing iPSCs maintained its pluripotency in the stem cell stage with genetic modification before differentiation. Furthermore, we investigated the CAR expression in the differentiated cells. Anti-EpCAM CAR expression could be significantly detected in differentiated cells by RT-PCR (Fig. 3B). This CAR expression was also shown by flow cytometry assay with 81% in CAR-expressing iPSCs and 74.2% in CAR-expressing differentiated iNK cells (Fig. 3C), which was maintained after the 47-day differentiation. However, GFP expression was reduced from 64% in CAR-expressing iPSCs to merely 1.5% in CAR-expressing differentiated iNK cells (Fig. 3D). This was consistent with the morphological observation alongside the 47-day differentiation (Fig. 2B).

**Fig. 4:**
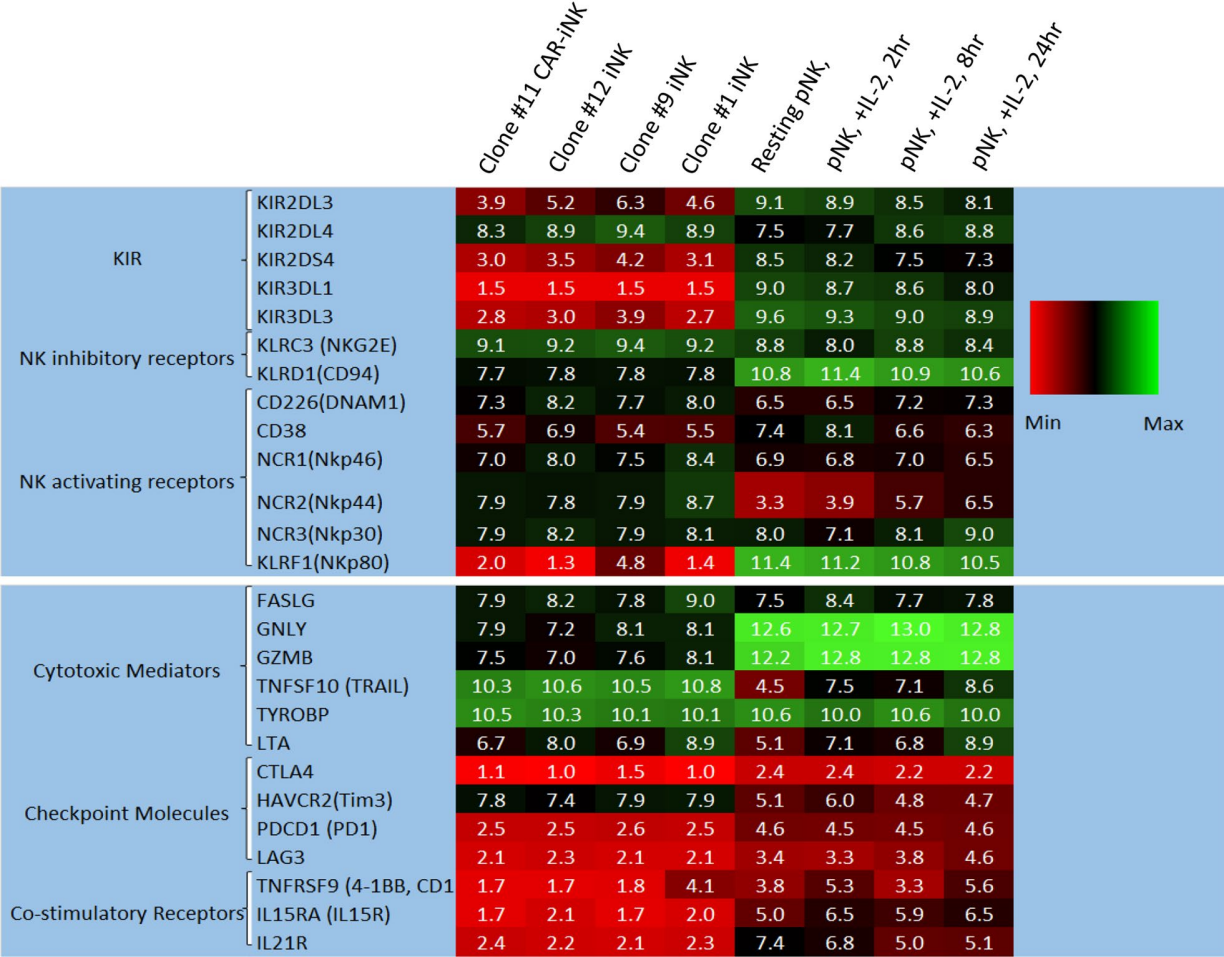
Heat map comparison of mRNA expression between unmodified and CAR-expressing iNK cells, as well as pNK cells. Clone #11 CAR-iNK cells generated in this study and Clone #12-, Clone #9- and Clone #1-iNK cells from our previous study [11] were used. For comparison purpose, additional raw data files for pNK cells (GSE8059) were downloaded from the National Centre for Biotechnology Information (NCBI) repository Gene Expression Omnibus (GEO) database. The score magnitudes are shown

To verify the phenotype of these differentiated cells, we profiled the mRNA expression pattern of unmodified and CAR-expressing iNK cells using a microarray assay (Fig. 4). Heat-map comparison of the expression of the important receptors of NK cells, including KIRs, inhibitory receptors, and activating receptors, demonstrated an overall similarity between the two types of iNK cells. They also showed comparable expression profiles for key functional molecules including cytotoxic mediators, checkpoint molecules and co-stimulatory receptors. In consistent with our previous results [11], the transcription of many KIR genes in iNK cells, including CAR-expressing iNK cells, was close to undetectable except the framework KIR gene KIR2DL4, whereas all examined KIR genes were actively expressed in pNK cells. Another obvious difference in mRNA expression between iNK cells and pNK cells was NKp80, an activating receptor of NK cells, with close to undetectable values in iNK cells and high expression values in pNK cells. The expression patterns of the cytotoxic mediator TRAIL were opposite, showing high expression values in iNK cells but low expression values in pNK cells.

**Fig. 5:**
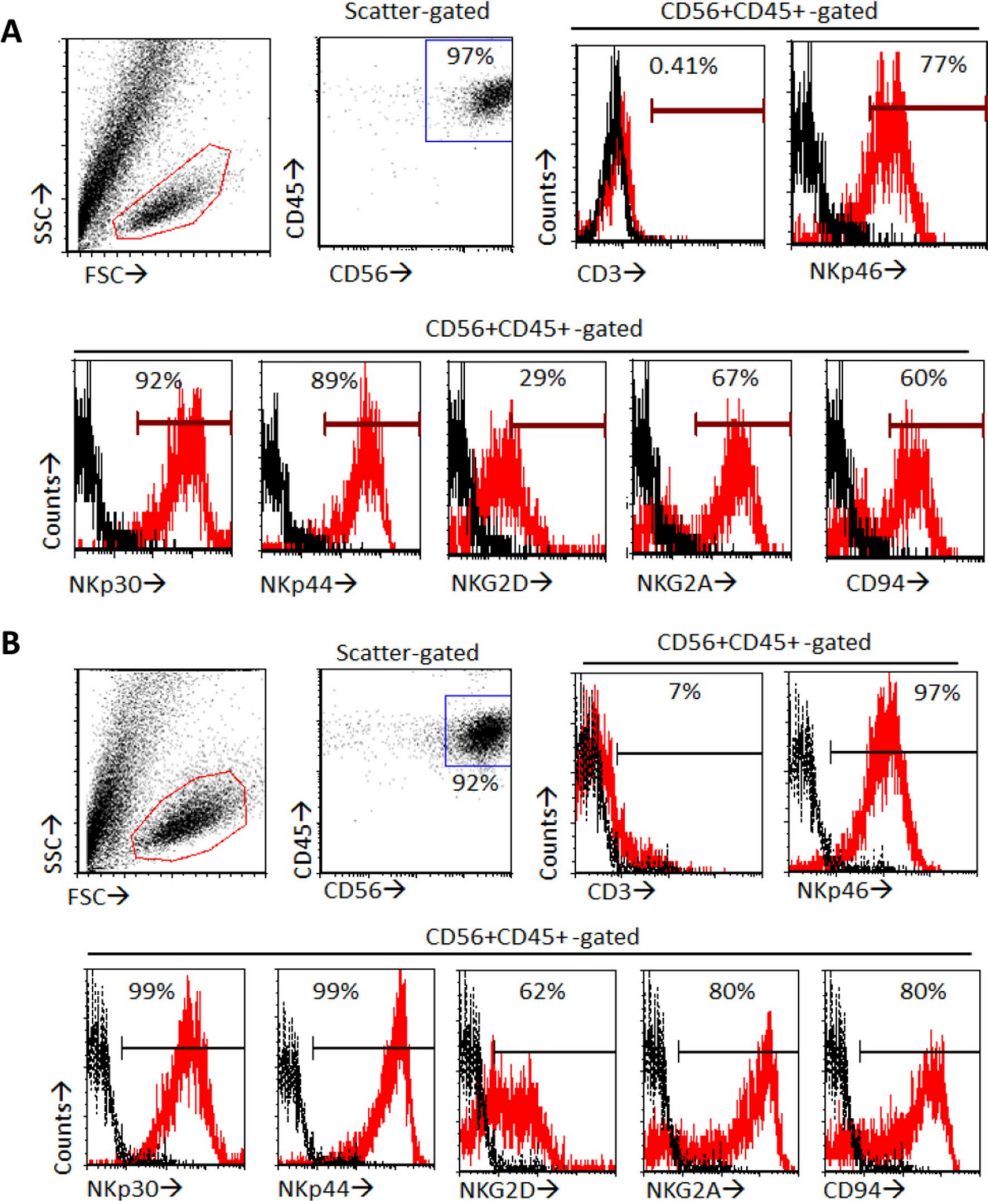
Phenotyping of iNK and αEpCAM CAR iNK. Differentiated iNK cells (**A**) and EpCAM CAR iNK cells (**B**) collected on Day 47 were analysed through flow cytometry. Black and red histograms represent isotype control antibody- and specific antibody-stained cells, respectively

We further performed flow cytometric analysis of NK cell markers to unmodified and CAR-expressing iNK cells. CAR-expressing iNK cells shared similar NK-like phenotype with unmodified iPSC-derived iNK cells, as a high purity of over 92% CD45+CD56+CD3- population (Fig. 5). Flow cytometric analysis further confirmed that these iNK cells expressed numerous NK cell receptors including natural cytotoxicity receptors (NKp46, NKp30 and NKp44), activating receptor (NKG2D), and inhibitory receptors (CD94, NKG2A). CAR-expressing iNK cells had a relatively high expression of the NK receptors at the protein level compared to the unmodified ones (Fig. 5). Hence, we confirmed that the iNK cells derived from CAR-expressing iPSCs displayed a NK cell-like phenotype with anti-EpCAM CAR expression.

### Cytotoxicity of anti-EpCAM CAR iNK cells against cancer cells with little on-target off-tumour effects

**Fig. 6:**
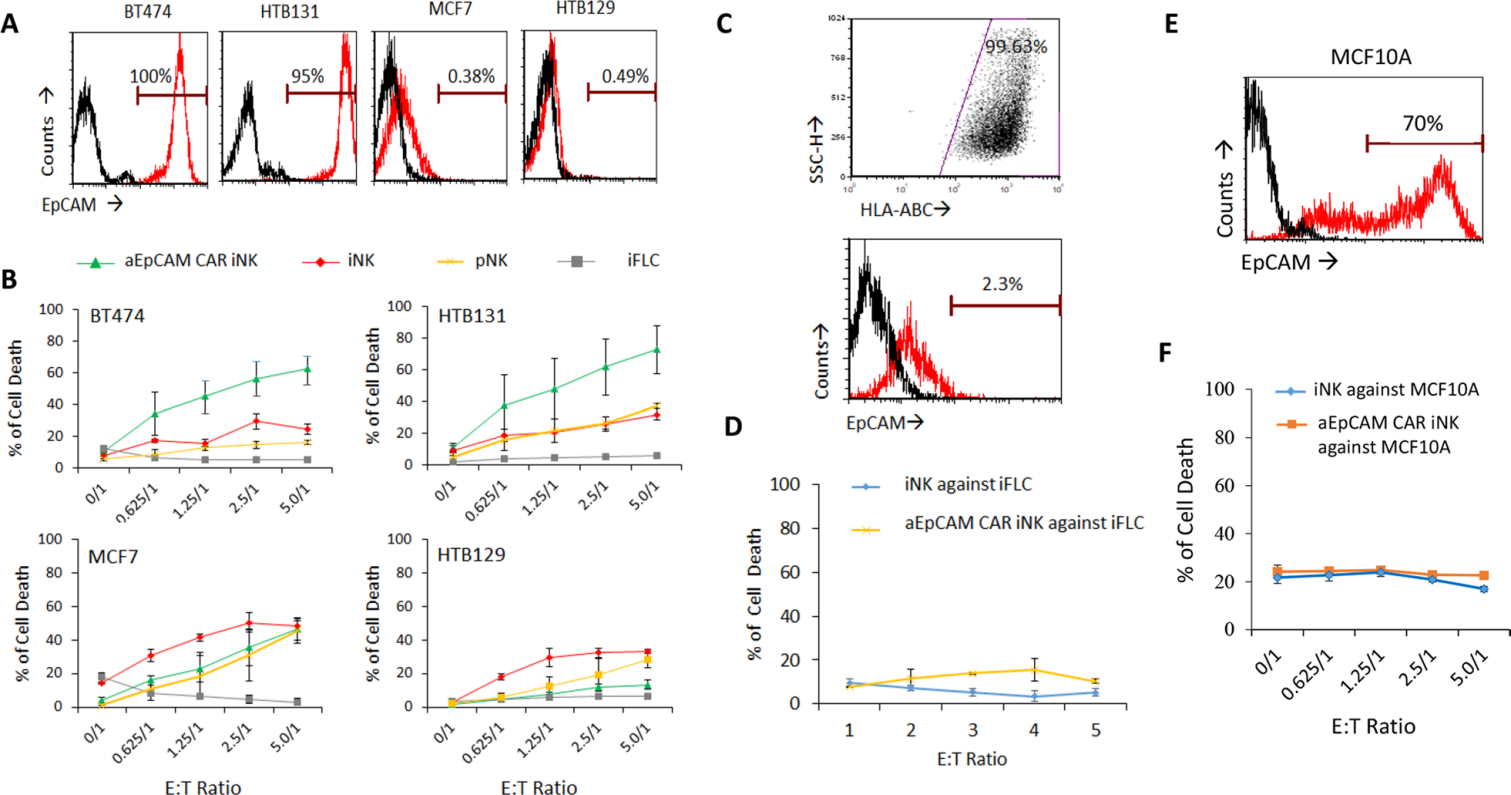
Cell lytic effects of anti-EpCAM CAR iNK cells against cancer cells. **A** EpCAM expression on human breast cancer lines BT474, HTB131, MCF7 and HTB129. Black and red histograms represent isotype- and anti-EpCAM antibody-stained cells, respectively. **B** anti-EpCAM CAR iNK cells displayed increased cytotoxicity against EpCAM-positive cancer cell line BT474 and HTB131. The data are presented as the mean ± SD of three repeated experiments. **C** Expression of HLA class 1 molecules and EpCAM on iPSC-derived FLC. **D** Autologous iFLC cells are resistant to iNK- or CAR-iNK-mediated cytotoxicity. The data are presented as the mean ± SD of three repeated experiments. **E** Expression of EpCAM on normal human breast epithelial cell MCF10A. **F** anti-EpCAM CAR iNK cells showed little cytotoxicity against normal human breast epithelial cell MCF10A. The data are presented as the mean ± SD of three repeated experiments

After observing the clear expression of NK receptors and CAR on iNK cells, we subsequently performed killing assay to study the cytotoxicity of these CAR-expressing iNK cells against tumour targets. Since an anti-EpCAM CAR was introduced in the iNK cells, cancer cell lines with EpCAM expression (BT474 and HTB131) and without EpCAM-expression (MCF7 and HTB129) were chosen as targets for this cytotoxicity assay (Fig. 6A). Besides the CAR-expressing iNK cells, unmodified iNK cells and primary NK cells (pNK) were also included as effector cells. When targeting EpCAM positive targets BT474 and HTB131, as expected, CAR-expressing iNK cells showed higher dose-dependent cytolytic effects than unmodified iNK and pNK cells, from an effector: target ratio of 0.625:1–5:1. When targeting EpCAM negative cells, CAR-expressing iNK cells shared similar cytolytic effects with unmodified iNK and pNK cells (Fig. 6B). These results demonstrated a strong EpCAM-specific cytotoxicity of anti-EpCAM CAR-expressing iNK cells against cancer cells.

Given the unique feature of NK cell activation in the tolerance to the normal cells [2], we further examined whether CAR gene incorporation would affect the iNK cell tolerability. Parental iPSC-derived fibroblast-like cells (iFLCs) were used as the target of autologous cells. iFLCs showed intact expression of HLA-A, B, C and a low expression of EpCAM antigens by flow cytometry assay (Fig. 6C). Both CAR-expressing iNK cells and unmodified iNK cells did not kill these iFLCs (Fig. 6D), indicating the tolerance of CAR-expressing iNK cells to autologous healthy cells. In addition, we evaluated the on-target off-tumour possibility of CAR-expressing iNK cells by targeting a normal human breast epithelial cell, MCF10A. Even with the 70% of EpCAM expression on MCF10A (Fig. 6E), neither CAR-expressing iNK cells nor unmodified iNK cells exhibited strong cytotoxicity against this normal epithelial cell line (Fig. 6F), which confirmed the ability of the iNK cells generated in this study in distinguishing normal cells from malignant ones. All together we demonstrated that the EpCAM-specific cytotoxicity of anti-EpCAM CAR-expressing iNK cells against tumour cell lines, with tolerance towards autologous normal cells and little on-target off-tumour effects.

## Discussion

NK cells are notorious for being difficult in genetic engineering. With low level expression of low-density lipoprotein receptor, NK cells are hardly transduced by lentiviral vector [29], a common process in CAR-T generation, only yielding a small percentage of CAR-expressing NK cells. Electroporation is another choice to genetically engineer NK cells. Most studies focused on mRNA-based electroporation which only provided transient expression of CAR on NK cells [23, 30, 31]. NK cells are difficult to be modified with DNA-based electroporation or transduced by commonly used lentiviral or retroviral vectors, and low transfer efficiencies are often observed [32]. Thus, genetic modification of primary NK cells for CAR expressing is a key obstacle down the road of CAR-NK application in cancer immunotherapy. The current study, for the first time, validated a method of generating CAR-NK cells through a site-specific integration of a CAR expression cassette in iPSCs, followed by NK cell differentiation from the modified iPSCs.

Using a ZFN/AAVS1 system that we previously developed and validated for specific loading of gene expression cassette in the iPSC genome [18, 33], in the current study, we genetically engineered the human iPSCs precisely to express an anti-EpCAM CAR by ZFNs-mediated AAVS1 site-specific integration. The donor sequence of anti-EpCAM CAR expression cassette was efficiently introduced into the iPSC genome and precisely in AAVS1 locus. Among the five examined clones, we identified one with biallelic AAVS1 site integration and three clones with monoallelic AAVS1 site integration, which was a quite high percentage of successful genetic modification. Furthermore, no random integration of donor sequence, other than AAVS1 site, was found in the iPSC genome by Southern blot. This verified the high fidelity and efficiency of gene editing by ZFNs in AAVS1 site, which is an unignorable advantage of this nuclease comparing to the popular CRIPSR/Cas9 system. Although CRISPR/Cas9 is flexible and versatile in genome editing, it often comes with a high off-target risk when dealing with site-specific integration [34, 35].

We previously developed a 47-day differentiation protocol to generate NK cells (iNK) from iPSCs [11]. Here, we adopted this protocol to differentiate the genetic modified iPSCs to generate CAR-iNK cells. Consistent with our previous report, even modified with CAR expression cassette, iNK cells were successfully derived according to this protocol. The yield was comparable between unmodified iPSCs and CAR-expressing iPSCs [11]. iNK cells from CAR-expressing iPSCs were morphologically similar to primary NK cells, displaying CD45+CD56+CD3- phenotype and NK activating and inhibitory receptors. The genetic modification in AAVS1 site did not disturb the iPSC-to-iNK differentiation and exhibited no effect on the morphology of iNK cells. This was in line with the stable expression of transgene in iPSCs derivatives after genetic modification in AAVS1 site [36, 37].

We further demonstrated the anti-EpCAM CAR expression on iNK cells derived from CAR-expressing iPSCs. EpCAM CAR expression was relatively weak in some of the clones in pluripotent stage (Fig. 1E). This could be the result of silence or down-regulation of the CMV promoter-driving expression in those clones [38–40], while the CMV promoter-driving expression could be much stronger in differentiated cells [41]. On the other hand, we observed the decrease in GFP expression alongside the differentiation of iPSC to iNK cells. This decrement in GFP could be explained by decreased EF1α promoter-driving expressing in differentiated cells compared to its expression in pluripotent stem cells [28]. Minimizing the risk of transgene silencing is one of our motivations in this study to adopt the approach of site-specific integration into a genomic safe harbour locus, in which the inserted gene expression cassette functions predictably. However, it has been shown that transgene silencing in the AAVS1 locus of iPSC-derived cells can still occur if weaker cell type-specific promoters are used [40]. The observed decrease in GFP expression in iNK cells, which was under the control of the human EF1a cellular promoter, is consistent with the previous report. The expression of EpCAM CAR was under the control of the strong viral CMV promoter, which has been successfully used to drive transgene expression in primary human NK cells [42]. We did not observe an obvious decrease of the CAR expression when iPSCs differentiate into iNK cells. Indeed, the activities of both EF1a and CMV promoters introduced by lentiviral transfer decreased during embryoid body differentiation of human embryonic stem cells (hESCs) [43]. The discrepancy between the previous study and our results was possibly related to the genomic locus in which an expression cassette was inserted (AAVS1 in this study and random integration when lentiviral vectors are used), differentiation procedure, or both. Further detailed research is needed to clarify these issues. Nevertheless, our results indicate that EF1α-driving expression would be more efficient in facilitating the selection of iPSCs, while a CMV promoter-containing cassette would be more suitable for driving transgene expression in differentiated cells.

With the expression of anti-EpCAM CAR, these CAR-expressing iNK cells displayed significantly higher cytotoxicity towards NK resistant, EpCAM-positive cancer cell lines BT474 and HTB131 in comparison with primary NK and unmodified iNK cells. Such cytotoxicity was antigen dependent as no obvious killing was found against EpCAM-negative cancer cell lines MCF7 and HTB129. Importantly, we detected no significant cytolytic effect of these CAR-expressing iNK cells on EpCAM-expressing autologous fibroblasts derived from the parental iPSCs (iFLCs) and an EpCAM-positive allogenic normal breast epithelial cell line, MCF10A. NK cells express a panel of structurally distinct, germline-encoded activating receptors such as NKG2D and inhibitory receptors such as killer Ig-like receptors (KIRs) and NKG2A. The balance of activating and inhibitory signalling determines the reactivity of NK cells and is crucial for NK cells to differentiate normal and abnormal cells [2, 44]. Since normal cells express HLA class I molecules, the cognate ligands of NK cell inhibitory receptors, but no or a low level of NKG2D ligands, the overall level of inhibitory receptor signalling outweighs activating receptor signalling and NK cell activation is blocked, resulting in the tolerance of NK cells to the normal cells. Considering that iNK cells are known to display variable expression patterns of KIRs [45] and it was unclear whether our iNK cells, especially EpCAM CAR-armed iNK cells, were able to display such a tolerance towards normal cells, we specifically designed the above experiments to examine the issue. Both iFLCs and MCF10A cells express MHC class I molecules (Fig. 6C) [46]. While the EpCAM antigen of the two types of normal cells interacted with the EpCAM CAR to activate iNK cells, the MHC class I molecules could bind to the inhibitory receptors, such as CD94 and NKG2A (Fig. 5A), of the same iNK cells. No killing effects against iFLCs and MCF10A cells by the EpCAM CAR-iNK cells indicated that the inhibitory signalling overrode the activating signalling and the activation of the CAR-iNK cells was somehow inhibited. Thus, our results demonstrated that the regulation of NK cell activity is subject to an array of activating and inhibitory receptors including a transferred CAR. This observation is consistent with a recent report that shows that, unlike HER2 CAR-T cells, HER2 CAR-NK cells do not elicit enhanced cytotoxicity against HER2-positive non-malignant human lung epithelial cells due to the recognition of MHC class I molecules [47]. Our observation further demonstrated that similar to what happens in NK cells, in iNK cells, the integration of the activating and inhibitory signals could also determine the magnitude of CAR-NK cell reactivity.

After examining the phenotype and function of CAR-expressing iNK cells in vitro in the current proof-of-concept study, further studies should be conducted to evaluate the efficacy and persistence of these CAR-expressing in vivo with an animal model. Also, the distribution and kinetics of the iNK cells shall be fully understood to improve this CAR-expressing iNK-based cancer therapy. In addition, in vitro expansion of iNK cells maybe with feeder cells could also be investigated to enhance the production of therapeutic iNK cells. Nevertheless, the preliminary results obtained in this study are highly encouraging and pave way for adopting such a site-specific modification approach in iPSCs for large-scale generation of CAR-expressing iNK cells. We believe this would significantly broaden the applicability of CAR-NK cell therapy.

## Conclusion

This study generated anti-EpCAM CAR-expressing iPSC clones by ZFNs-mediated AAVS1 site-specific integration, which was seamless and efficient. CAR-expressing iNK cells were subsequently derived from the modified iPSCs and were shown to display a NK cell-like phenotype with the expression of activating and inhibitory receptors, apart from an additional potent expression of anti-EpCAM CAR. The CAR-expressing iNK cells exhibited the antigen-dependent cytotoxicity against EpCAM-positive tumours cells, but not against EpCAM-positive normal cells, demonstrating to be a safe CAR-effector cell source for immunotherapy against cancer. Our approach of site-specific modification on iPSCs to generate CAR-expressing iNK cells provides a novel solution for the development of CAR-iNK cells.

## Supplementary Information

**Additional file 1**. Material and Methods, and Supplemental Figures 1–4.

## Data Availability

Not applicable.

## Abbreviations

AAVS1: Adeno-associated virus integration site 1
CAR: Chimeric antigen receptor
CMV: Cytomegalovirus
DLL1: Notch ligand Delta-like-1
EF1α: Eukaryotic translation elongation factor 1 alpha
EpCAM: Epithelial cell adhesion molecule
GFP: Green fluorescence protein
iFLC: IPSC-derived fibroblast-like cell
iNK: IPSC-derived NK cell
iPSC: Induced pluripotent stem cell
KIR: Killer-cell immunoglobulin-like receptor
Neo: Neomycin resistance
NK: Natural killer cell
PBMC: Peripheral blood mononuclear cell
PGK: Phosphoglycerate kinase 1
pNK: Primary NK cell
scFv: Single-chain variable fragment
ZFN: Zinc finger nuclease
