# Supplementary material for: Targeted integration of EpCAM-specific CAR in human induced pluripotent stem cells and their differentiation into NK cells
Source: Stem Cell Res Ther. 2021 Nov 21;12:580. doi: 10.1186/s13287-021-02648-4 (PMC8607711; doi:10.1186/s13287-021-02648-4)
Supplement: Supplementary file 1 — Additional file 1. Material and Methods, and Supplemental Figures 1–4. [file 13287_2021_2648_MOESM1_ESM.pdf]

Targeted Integration of EpCAM-specific CAR in Human Induced Pluripotent Stem Cells and Their Differentiation into NK Cells

Shin Yi Tang, Shijun Zha, Zhicheng Du, Jieming Zeng, Detu Zhu, Yumei Luo, and Shu Wang

SUPPLEMENTAL INFORMATION

Material and Methods

NK cell differentiation from iPSCs

A two-stage protocol was used to generate iNK cells from iPSCs. Before starting iPSC differentiation, OP9-DLL1, a modified OP9 cell line expressing Notch delta-like ligand 1 (DLL1), were cultured on 0.1% gelatin (StemCell Technologies, Vancouver, BC, Canada)-coated T75 flasks for 9 days, with half of the OP9 medium (MEMα, Gibco, Waltham, MA and 20% FBS, Hyclone, Logan, UT, Catalogue No: SV30160.03, Lot No.: RE00000006) changed every 3-4 days till confluence. In the first stage of the differentiation, iPSCs (1-1.5 x 10^6) were seeded on the overgrown OP9-DLL1 cells for 12 days to derive hematopoietic progenitor cells, with the half medium changed every 4 days. In the second stage, the iPSC/ OP9-DLL1 cells were harvested using 1 mg/ml collagenase IV (StemCell Technologies) and TrypLE Express (Gibco). The OP9-DLL1 cells and cell clumps were removed by plastic adherence for 45 minutes followed by 100 µM cell strainers (BD Biosciences, Franklin Lakes, NJ). The remaining non-adherent cells were then cultured in a T75 flask pre-coated with OP9-DLL1 in the OP9 medium supplemented with 10% FBS, 10 ng/ml SCF (PeproTech, Rocky Hill, NJ), 5 ng/ml Fms-related tyrosine kinase 3 ligand (FLT3L, Peprotech), 5 ng/ml IL-7 (Peprotech) and 10 ng/ml IL-15 (Peprotech) for 7 days. After that, the differentiated cells were harvested using Versene (Thermo Fisher Scientific) and seeded at a density of 2-3 x 10^5 cells on each well in a six-well plate that was pre-coated with OP9-DLL1 cells. This procedure was repeated on a weekly basis for another 3–4 weeks. The finally harvested cells were purified by density gradient centrifugation using Ficoll-Paque PLUS (GE Healthcare Life Sciences, Milwaukee, WI).

Microarray analysis

The total RNA from Clone #11 CAR-iNK cells, Clone #12-, Clone #9-, and Clone #1-iNK cells (from our previous study) were isolated with TRIZOL reagent (Invitrogen Life Technologies, Carlsbad, CA). RNA quality was assessed with the Agilent Model 2100 Bioanalyzer (Agilent Technologies, Palo Alto, CA) . Total RNA (150 ng) was used to generate complementary RNA which was then biotinylated, fragmented and hybridized to the GeneChip Human Genome 2.0 ST Array (Affymetrix, Thermo Fisher, Waltham, MA). The arrays were washed, stained, and scanned using the Affymetrix Model 450 Fluidics Station and Affymetrix Model 3000 7G scanner. All procedure was performed according to manufacturer's protocol using the microarray facility at Institute of Molecular & Cell Biology (IMCB), A*STAR, Singapore. For comparison purpose, additional raw data files were downloaded from the National Center for Biotechnology Information (NCBI) repository Gene Expression Omnibus (GEO) database: NK cells (GSE8059), γδ T cells (GSE 27291), resting CD8 T cells (GSE8059), B cells (GSE12195), and CD4 T cells (GSE 15659). To allow comparison across arrays, expression values were normalized

with robust multi-array average (RMA) using the Affymetrix Expression Console software. The percentage of genes analysis of variance (ANOVA) expressed on array was calculated using the number of probe sets labelled present or marginal based on an applied algorithm. Subsequently, comparisons on selected genes were analyzed using the Affymetrix Transcriptome Analysis Console software for cluster analysis and relative gene expression. The comparison between different arrays dataset was done using Galaxy software.

atgcttctcctggtgacaagccttctgctctgtgagttaccacacccagcattcctcctgatcccaggcgcgcatgccgatatccagatgaccc
agtccccgtcctccctgagtgcttctgttggtgaccgtgttaccatcacctgccgttccaccaaatccctcctgcactccaacggtatcacctac
ctttattggtatcaacagaaaccgggtaaagctccgaaacttctgatctaccagatgtccaacctggcttccggtgttccgtctcgtttctccag
ttctggttctggtaccgacttcaccctgaccatctcttctctgcagccggaagacttcgctacctactactgcgctcagaacctggaaatcccgc
gtaccttcggtcagggtaccaaagttgaacttaagcgcgctaccccgtctcacaactcccaccaggttccatccgcaggcggtccgactgcta
actctggaactagtggatccgaagtacagctggttcagtccggcccgggtcttgttcaaccgggtggttccgttcgtatctcttgcgctgcttct
ggttacacgttcaccaactacggcatgaactgggtcaaacaggctccgggtaaaggcctggaatggatgggctggatcaacacctacaccg
gtgaatccacctacgctgactccttcaaaggtcgcttcactttctccctcgacacaagtgctagtgctgcatacctccaaatcaactcgctgcgt
gcagaggatacagcagtctattactgcgcccgtttcgctatcaaaggtgactactggggtcaaggcacgctgctgaccgtttcctcggctagc
ttcgtgccggtcttcctgccagcgaagcccaccacgacgccagcgccgcgaccaccaacaccggcgcccaccatcgcgtcgcagcccctgtc
cctgcgcccagaggcgtgccggccagcggcggggggcgcagtgcacacgagggggctggacttcgcctgtgatatctacatctgggcgcc
cttggccgggacttgtggggtccttctcctgtcactggttatcaccctttactgcaaccacaggaacaggagtaagaggagcaggctcctgca
cagtgactacatgaacatgactccccgccgccccgggcccacccgcaagcattaccagccctatgccccaccacgcgacttcgcagcctatc
gctcccgtttctctgttgttaaacggggcagaaagaagctcctgtatatattcaaacaaccatttatgagaccagtacaaactactcaagagg
aagatggctgtagctgccgatttccagaagaagaagaaggaggatgtgaactgagagtgaagttcagcaggagcgcagacgcccccgcgt
accagcagggccagaaccagctctataacgagctcaatctaggacgaagagaggagtacgatgttttggacaagagacgtggccgggacc
ctgagatgggggaaagccgagaaggaagaaccctcaggaaggcctgtacaatgaactgcagaaagataagatggcggaggcctacag
tgagattgggatgaaaggcgagcgccggaggggcaaggggcacgatggcctttaccagggtctcagtacagccaccaaggacacctacg
acgcccttcacatgcaggccctgccccctcgctga

Colour code label:
Signal peptide; FMC63-scFv; CD8 Hinge and Transmembrane domain; CD28; 4-1BB; CD3zeta.

Supplemental Figure 1. Sequence data of EpCAM CAR construct.

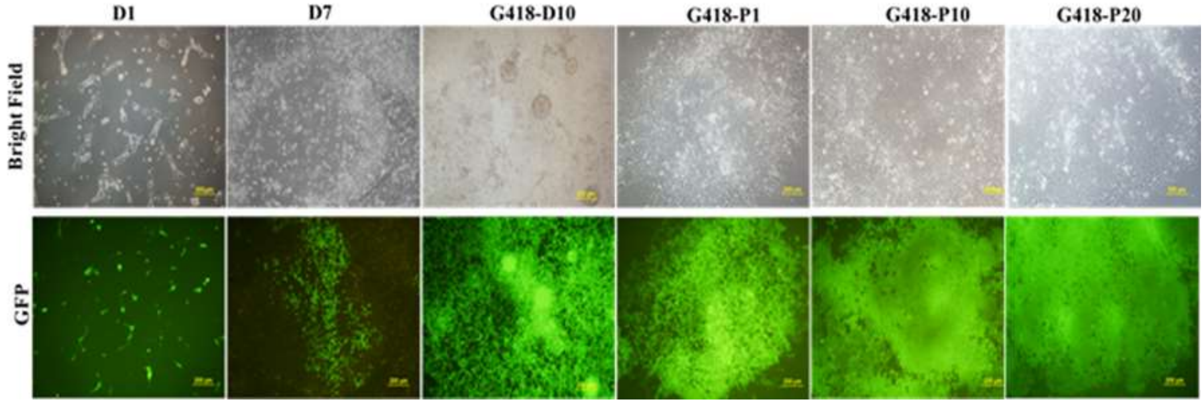

Supplemental Figure 2. Enrichment of the modified iPSCs and the stability of GFP-positive iPSCs. Manual selection of GFP-positive cell clumps was first performed one week post-nucleofection, followed by continuous Geneticin (G418) drug selection. Images were taken on Day 1 and Day 7 post-nulecofection, 10 days after G418 selection, G418-resistant iPSC colonies at passages 1, 10 and 20.

A. ACCTTCNGTNCTTTGGAGGTCTACTGAGGTTCTGCGACGANGTACTGGGCCACATGTTGTCCCCTGCCCAGGGAGGTCCTGCTTT
CTCTGACCTGCATTCTCTCCCCTGGGCCTGTGCCGCTTTCTGTCTGCAGCTTGTGGCCTGGGTCACCTCTACGGCTGGCCCAGATC
CTTCCCTGCCGCCTCCTTCAGGTTCCGTCTTCCTCCACTCCCTCTTCCCCTTGCTCTCTGCTGTGTTGCTGCCCAAGGATGCTCTTTC
CGGAGCACTTCCTTCTCGGCGCTGCACCACGTGATGTCCTCTGAGCGGATCCTCCCCGTGTCTGGGTCCTCTCCGGGCATCTCTCC
TCCCTCACCCAACCCCATGCCGTCTTCACTCGCTGGGTTCCCTTTTCCTTCTCCTTCTGGGGCCTGTGCCATCTCTCGTTTCTTAGGA
TGGCCTTCTCCGACGGATGTCTCCCTTGCGTCCCGCCTCCCCTTCTTGTAGGCCTGCATCATCACCGTTTTTCTGGACAACCCCAAA
GTACCCCGTCTCCCTGGCTTTAGCCACCTCTCCATCCTCTTGCTTTCTTTGCCTGGACACCCCGTTCTCCTGTGGATTCGGTCACC
TCTCACTCCTTTCATTTGGGCAGCTCCCCTACCCCCCTTACCTCTCTAGTCTGTGCTAGCTCTTCCAGCCCCCTGTCATGGCATCTTC
CAGGGGTCCGAGAGCTCAGCTAGTCTTCTTCCTCCAACCCGGGCCCCTATGTCCACTTCAGGACAGCATGTTTGCTGCCTCCAGG
GATCCTGTGTCCCCGAGCTGGGACCACCTTATATTCCCAGGGCCGGTTAATGTGGCTCTGGTTCTGGGTACTTTTATCTGTCCCCTC
CACCCCACAGTGGGGCCACTAGGGACAGGATTGGTGACAGAAAAGCCCCATCCTTAGGCCTCCTCCTTCCTAGTCTCCTGATATT
GGGTCTAACCCCCACCTCCTGTTAGGCAGATTCCTTATCTGGTGACACACCCCCATTTCCTGGAGCCATCTCTCTCCTTGCCAGAAC
CTCTAAGGTTTGCTTACGATGGAGCCAGAGAGGATCCTGGGAGGGAGAGCTTGGCAGGGGTGGAAGGAAAGGGG

B.

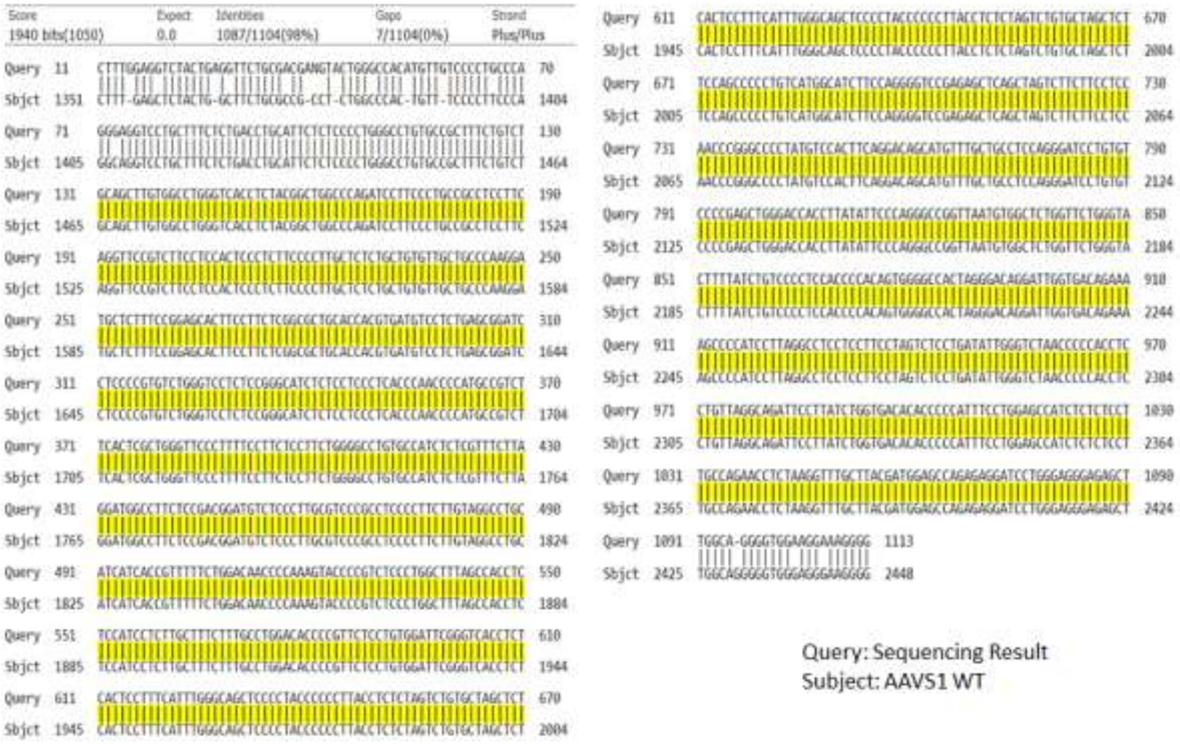

Query: Sequencing Result
Subject: AAVS1 WT

C.

AAACTACGGCNTTTGAGCGTCTACTAGGCTTCTGCGCCGCCTCTGGCCCACTGTATTCCCGCTTCCCAGGCAGGTCCTGCTTT
CTCTGACCTGCATTCTCTCCCCTGGGCCTGTGCCGCTTTCTGTCTGCAGCTTGTGGCCTGGGTCACCTCTACGGCTGGCCCAG
ATCCTTCCCTGCCGCCTCCTTCAGGTTCCGTCTTCCTCCACTCCCTCTTCCCCTTGCTCTCTGCTGTGTTGCTGCCCAAGGATGC
TCTTTCCGGAGCACTTCCTTCTCGGCGCTGCACCACGTGATGTCCTCTGAGCGGATCCTCCCCGTGTCTGGGTCCTCTCCGGG
CATCTCTCCTCCCTCACCCAACCCCATGCCGTCTTCACTCGCTGGGTTCCCTTTTCCTTCTCCTTCTGGGGCCTGTGCCATCTCT
CGTTTCTTAGGATGGCCTTCTCCGACGGATGTCTCCCTTGCGTCCCGCCTCCCCTTCTTGTAGGCCTGCATCATCACCGTTTTTC
TGGACAACCCCAAAGTACCCCGTCTCCCTGGCTTTAGCCACCTCTCCATCCTCTTGCTTTCTTTGCCTGGACACCCCGTTCTCCT
GTGGATTCGGGTCACCTCTCACTCCTTTCATTTGGGCAGCTCCCCTACCCCCCTTACCTCTCTAGTCTGTGCTAGCTCTTCCAGC
CCCCTGTCATGGCATCTTCCAGGGGTCCGAGAGCTCAGCTAGTCTTCTTCCTCCAACCCGGGCCCCTATGTCCACTTCAGGAC
AGCATGTTTGCTGCCTCCAGGGATCCTGTGTCCCCGAGCTGGGACCACCTTATATTCCCAGGGCCGGTTAATGTGGCTCTGGT
TCTGGGTACTTTTATCTGTCCCCTCCACCCCACAGTGGGGCGTCGACGGTATCGATAAGCTAGCTTGGGCTGCAGGTCGAGGG
ACCTAATAACTTCGTATAGCATACATTATACGAAGTTATATTAAGGGTTCCGGATCAGCTTGATGGGGATCCAGACATGATAAGA
TACATTGATGAGTTTGGACAAACCACAACTAGAATGCAGTGAAAAAAATGCTTTATTTGNGAAAATTGGGGATGCTATTGCTT
GATTTGAACCATAATAGGCTGCAA

D.

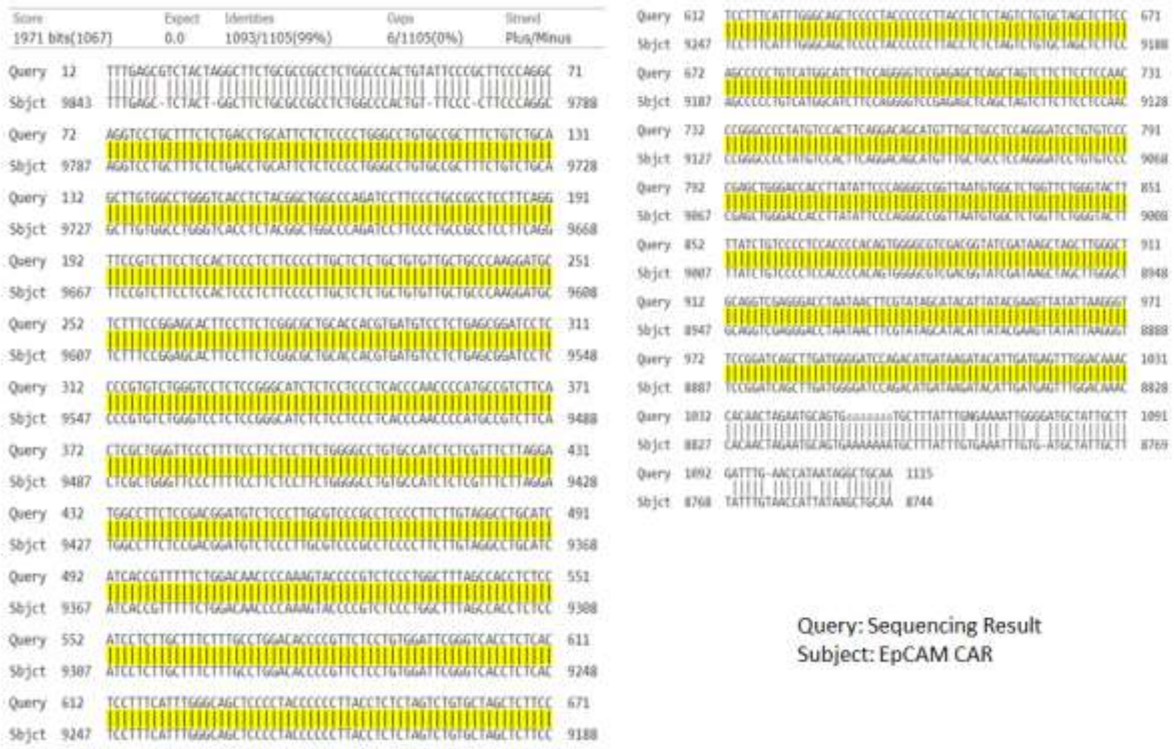

Supplemental Figure 3. Sequencing of PCR amplicons. A. Sequencing result of WT allele. PCR amplicon was amplified with FP1 and RP1. **B.** Alignment of the sequencing result with AAVS1 WT sequence. **C.** Sequencing result of EpCAM CAR allele. PCR amplicon was amplified by FP2 and RP1. **D**. Alignment of the sequencing result with EpCAM CAR sequence.

A **B**

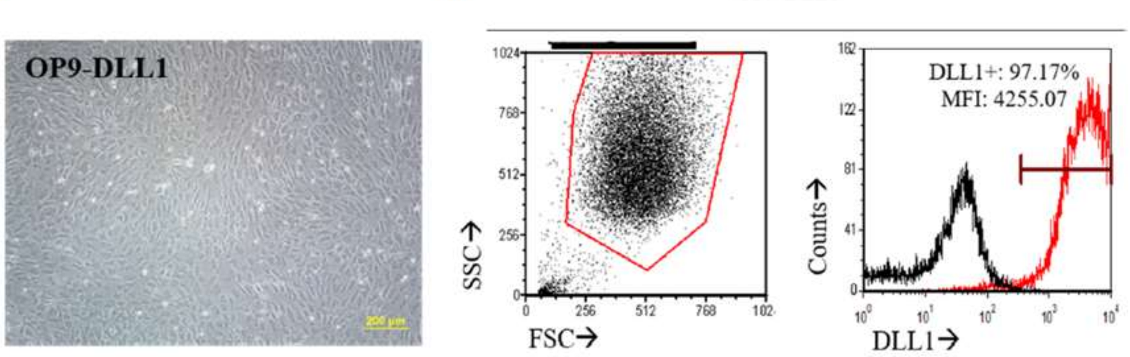

Supplemental Figure 4. OP9-DLL1 cells used for iNK cell differentiation from iPSCs. (**A**) Morphology of OP(-DLL1 cells. (**B**) OP9-DLL1 cells express a high level of DLL1. Around 97% of the cells were positively detected through flow cytometry. Black histogram represents an isotype antibody stained cells and red histogram represents the DLL1-positive cells.
